# Efficacy of cataract surgeries performed during blindness prevention programs in Chongqing, China: a multicenter prospective study

**DOI:** 10.1186/s12886-023-03082-1

**Published:** 2023-08-10

**Authors:** Yongguo Xiang, Xiaoqin Wang, Xiaochuan Cao, Fang Wei, Yu Chen, Jianchuan Ran, Zhengqin Long, Qunwu Tan, Zhenying Lai, Li Liu, Desheng Zhao, Liang Xiong, Bin Tang, Wenjuan Wan, Ke Hu

**Affiliations:** 1 Chongqing Medical University, Chongqing, People’s Republic of China; 2 People’s Hospital of Tongliang District, Tongliang District, Chongqing, People’s Republic of China; 3 Youyang County People’s Hospital, Youyang County, Chongqing, People’s Republic of China; 4 Qianjiang Central Hospital, Qianjiang District, Chongqing, People’s Republic of China; 5 Wushan County People’s Hospital, Wushan County, Chongqing, People’s Republic of China; 6 People’s Hospital of Dazu District, Dazu District, Chongqing, People’s Republic of China; 7 People’s Hospital of Jiangbei District, Jiangbei District, Chongqing, People’s Republic of China; 8 Chongqing Key Laboratory of Ophthalmology, Chongqing Branch (Municipality Division) of National Clinical Research Center for Ocular Diseases, The First Affiliated Hospital of Chongqing Medical University, Chongqing Eye Institute, Chongqing, People’s Republic of China

**Keywords:** Blindness prevention, Cataract surgery, Visual acuity, Visual function, Quality of life

## Abstract

**Objective:**

To determine the efficacy of cataract surgeries in blindness prevention programs in Chongqing.

**Methods:**

During February–December 2019, we prospectively enrolled 487 patients (592 eyes) undergoing cataract surgery during blindness prevention programs in 6 Chongqing district/county hospitals (experimental group) and 481 patients (609 eyes) undergoing cataract surgery in the First Affiliated Hospital of Chongqing Medical University (controls). Uncorrected visual acuity (UCVA), refractive status, best corrected visual acuity (BCVA), slit lamp examination, and visual function/quality of life (VF-QOL) questionnaire scores were evaluated preoperatively, and at 1 and 6 months postoperatively.

**Results:**

In the experimental group, UCVA, BCVA, and VF-QOL scores at 1 and 6 months were better than the preoperative values (*P <* 0.05), but lower than the control-group values (*P <* 0.05). Rates of good UCVA and BCVA outcomes (≤ 0.5 logMAR) in the experimental group were 76.2% and 87.6%, respectively, at 1 month and 68.9% and 83.1%, respectively, at 6 months. Most eyes in the experimental (82.1%) and control (89.5%) groups had refractive errors within ± 1 D at 1 month. At 6 months, posterior capsule opacification (PCO) was more common in the experimental group (20.9% vs. 15.0%, *P* < 0.05). At 6 months, the main causes of visual impairment (UCVA > 0.5 logMAR) in the experimental group were uncorrected refractive errors (33.0%), PCO (29.5%), and fundus diseases (33.9%).

**Conclusion:**

Cataract surgeries in blindness prevention programs in Chongqing significantly improved visual acuity, VF, and QOL, but underperformed compared to surgeries in the tertiary teaching hospital.

## Background

The Blindness and Vision Impairment data of the Global Burden of Disease Study 2019 showed that globally, cataract remained the largest contributor to blindness in adults aged 50 years and older in 2020, affecting over 15 million individuals globally [1]. Cataract is also the leading cause of blindness in China [2, 3]. It is reported that more than one in five Chinese people between the ages of 45 and 89 years were affected by cataract during 1990–2015. As the population ages, cataract cases in China are expected to more than 240.83 million by 2050, and resulting in a significant socioeconomic burden [4].

Cataract phacoemulsification is currently the mainstream operation for cataract treatment. A series of national cataract blindness prevention projects such as Sight First China Action and One Million Poor Cataract Patients Restoring Vision have been successively implemented, resulting in remarkable achievements in the prevention and treatment of cataract blindness in China [5]. The cataract surgical coverage for patients with cataract-related severe visual impairment or blindness reached 62.7% in 2014 [6], and the cataract surgery rate (CSR) per million population increased from 370 to 2000 to 2205 in 2017 [2, 7]. However, China still lags behind developed countries in the treatment of cataract. In France, for example, the CSR reached 110,800 in 2012 [8]. Some other developing countries have also achieved better results than China, such as India with a CSR of 6000 in 2012 [9], Egypt with a CSR of 3674 in 2014 [10], and Iran with a CSR of 6328 in 2010 [11]. In addition to increasing the CSR, it is important to improve the efficacy of cataract surgery, especially in blindness prevention programs [12, 13]. However, an investigation showed that the comprehensive ophthalmic service capacity of county-level public hospitals in China is inadequate, and varies across regions [14, 15]. The ability to perform cataract surgery varies among ophthalmologists, and the average number of cataract surgeries performed annually is insufficient [14]. Especially in western China, the number of ophthalmologists and cataract surgeons per 50,000 population was only 1.10 and 0.43, respectively, in 2014 [16].

Chongqing is the largest municipality directly under the Central Government in China, it is located in southwest China, and is divided into 38 districts and counties. The socioeconomic level of different districts and counties is quite variable, and the condition of ophthalmic care in some hospitals is poor. According to the China Network of National Blindness Prevention and Treatment (http://www.moheyes.com/), the CSR of Chongqing reached 1,879 in 2017, just behind that of Shanghai, Tianjin, Jiangsu Province, and Sichuan Province [17], but the outcomes of cataract surgery remain unclear, especially cataract surgery performed during blindness prevention programs.

In this study, we followed up patients who underwent cataract surgery during blindness prevention programs in district and county hospitals in Chongqing in order to evaluate the long-term effects of such cataract surgeries and provide evidence for programs for the prevention and treatment of cataract blindness.

## Methods

### Patients

This multicenter prospective clinical study was conducted using regional sampling according to population distribution and GDP level (data were obtained from the National Bureau of Statistics of China; https://data.stats.gov.cn/index.htm). Patients who underwent cataract surgery during blindness prevention programs in 6 Chongqing hospitals (Jiangbei District People’s Hospital, Dazu District People’s Hospital, Tongliang District People’s Hospital, Qianjiang District Central Hospital, Youyang County People’s Hospital, and Wushan County People’s Hospital) between February and December 2019 were included as the experimental group. Patients who underwent cataract surgery in the First Affiliated Hospital of Chongqing Medical University during the same period were included as the control group. The following exclusion criteria were applied: (1) patients who were unable to complete the examination or questionnaire survey, and (2) patients with other vision-threatening ocular diseases (such as corneal leukoplakia, glaucoma, vitreous hemorrhage, retinal detachment, macular degeneration, and optic atrophy) detected during preoperative examination. This trial was conducted in accordance with the Helsinki Declaration, and was approved by the ethics committee of the First Affiliated Hospital of Chongqing Medical University, Chongqing, China. The trial is registered in the Chinese Clinical Trial Registry (registration no., ChiCTR1900022641). All the participants signed written informed consent forms before enrollment.

The main observation index was the incidence of posterior capsule opacification (PCO) in the study subjects. The estimated incidence of PCO was 30.0% in the experimental group and 20.0% in the control group. The probability of a type I error (alpha) was set at 0.05, and the probability of a type II error (beta) was set at 0.10 (i.e., the power was 90%). The test statistic used was the two-sided Z test with pooled variance (https://sample-size.net/). The required sample size was 392 eyes per group. Factoring in a loss to follow-up of 20% gave a final sample size of 942 eyes (471 eyes per group).

### Preoperative examination

All patients underwent a complete ophthalmological examination before the operation, including measurement of the uncorrected visual acuity (UCVA), subjective refraction, and best corrected visual acuity (BCVA); slit lamp examination, and fundus examination as well as cataract grading according to the Emery-Little classification. Contact ultrasound A-scan biometry combined with autokeratometry were used to measure ocular biological parameters in the experimental group, while the IOLMaster 500 was used in the control group (contact ultrasound A-scan biometry was used when the IOLMaster was unable to obtain the axial length). In the experimental group, the power of the intraocular lens (IOL) was determined using the SRK/T formula for eyes with an axial length > 22 mm and using the Hoffer-Q formula for eyes with shorter axial lengths. In the control group, the Haigis formula was used to calculate the IOL power. All patients filled in visual function (VF) and quality of life (QOL) questionnaires [18]. Visual acuity (VA) was expressed as the logarithm of the minimum angle of resolution (logMAR). On the basis of previous studies, we replaced a VA of counting fingers with 1.85 logMAR, hand motion with 2.30 logMAR, and light perception with 2.80 logMAR [19]. The visual outcomes of the operated eyes were graded as follows: good outcome, ≤ 0.5 logMAR; borderline outcome, > 0.5 logMAR to ≤ 1.0 logMAR; and poor outcome, > 1.0 logMAR [20].

### Surgical procedure

Phacoemulsification through a clear corneal incision was performed under topical anesthesia by 12 experienced surgeons in the experimental group and a single experienced surgeon in the control group. After phacoemulsification, the cortex was aspirated using coaxial irrigation/aspiration (an additional hydropolish technique was performed in the control group after the aspiration of the cortex [21]). Subsequently, the capsular bag was filled with a viscoelastic agent and a foldable posterior chamber IOL was implanted through an injector system. In cases of posterior capsular rupture, a anterior vitrectomy was performed, and the IOL was placed in the ciliary sulcus.

### Postoperative follow-up

The patients were followed up at 1 month and 6 months after the surgery. Complete eye examination was performed at each follow-up visit, including measurement of UCVA, subjective refraction, and BCVA, slit lamp examination, VF-QOL questionnaires, and PCO grade (according to Congdon et al. [22]). If necessary, a fundus examination was performed. Patients who did not attend the outpatient follow-up on time were contacted by telephone and administered the VF-QOL questionnaires. Both patients who completed the VF-QOL questionnaires in the outpatient clinic and on telephonic follow-up were included in the analysis.

### Consistency check

For the experimental group, 2 researchers were selected from each of the 6 participating hospitals, and all 12 researchers were trained in the research methods and passed the consistency test before the study began.

### Statistical analysis

The study results were expressed as mean and standard deviation or median and upper and lower quartiles [M (P25–P75)], as appropriate. The Kolmogorov-Smirnov test, *t*-test, Mann-Whitney *U* test, Kruskal-Wallis *H* test, χ^2^ test, and Fisher exact test were performed using SPSS software (version 22.0; SPSS Inc, Chicago, IL). *P*-values lower than 0.05 were considered statistically significant.

## Results

### General information

In total, we enrolled 519 subjects (635 eyes) in the experimental group and 532 subjects (675 eyes) in the control group. Of these, 32 subjects (43 eyes) in the experimental group and 51 subjects (66 eyes) in the control group were excluded because other vision-threatening ocular diseases were detected on preoperative examination or because the subjects were unable to complete the examination or questionnaire survey. Thus, 487 patients (592 eyes; 64.7% [315/487] women) were enrolled in the experimental group, with an average age of 69.04 ± 9.84 years, and 481 patients (609 eyes; 60.3% [290/481] women) were included in the control group, with an average age of 70.01 ± 9.58 years. There was no significant difference in sex, age, cataract type, and preoperative VF-QOL questionnaire scores between the two groups (*P* > 0.05; Table 1). However, the experimental group had worse preoperative UCVA and BCVA, a lower level of education, and a higher rate of hard nucleus cataract than the control group (*P* < 0.001; Table 1). Table 2 shows the number of cases and the rate of loss to follow-up after the surgery.

**Table 1:** Preoperative characteristics of patients in the experimental and control groups

	Experimental group	Control group	t/Z/χ^2^ value	*P* value
**Patients/Eyes**	487/592	481/609	N/A	N/A
**Sex** F	315 (64.7%)	290 (60.3%)	1.99	0.16
M	172 (35.3%)	191 (39.7%)		
**Age (years)**	69.04 ± 9.84	70.01 ± 9.58	1.550	0.12
**Education level**				
Elementary school or below	435 (89.3%)	246 (51.1%)^*^	178.807	< 0.001
Junior high school	50 (10.3%)	162 (33.7%)^*^		
High school or above	2 (0.4%)	73 (15.2%)^*^		
**Cataract type**				
Age-related	561 (94.8%)	569 (93.4%)	0.957	0.33
Other	31 (5.2%)	40 (6.6%)		
**Lens nuclear sclerosis**				
I–II	138 (23.3%)	211 (34.6%)^*^	24.355	< 0.001
III	294 (49.7%)	288 (47.3%)		
IV–V	160 (27.0%)	110 (18.1%)^*^		
**UCVA**	1.00 (0.69, 1.69)	0.92 (0.60, 1.30)	4.059	< 0.001
**BCVA**	0.82 (0.52, 1.30)	0.52 (0.30, 0.82)	11.76	< 0.001
**VF score**	48.48 (30.30, 60.61)	48.48 (27.27, 57.58)	0.479	0.63
**QOL score**	75.00 (61.11, 86.11)	75.00 (63.89, 83.33)	0.119	0.91

F, female; M, male; UCVA, uncorrected visual acuity; BCVA, best corrected visual acuity; VF, visual function; QOL, quality of life

^*^P < 0.05 vs. experimental group

**Table 2:** Number of cases and rate of loss to follow-up in the experimental and control groups

	Experimental group	Control group
**Total** (patients/eyes)	487/592	481/609
**1 month postoperatively**		
Outpatient follow-up (eyes)/Missing rate	453/23.5%	447/26.6%
VF-QOL questionnaire follow-up (patients)/Missing rate	425/12.7%	434/9.8%
**6 months postoperatively**		
Outpatient follow-up (eyes)/Missing rate	359/39.9%	373/38.8%
VF-QOL questionnaire follow-up (patients)/Missing rate	446/8.4%	453/5.8%

VF, visual function; QOL, quality of life

### VA

**Fig. 1:**
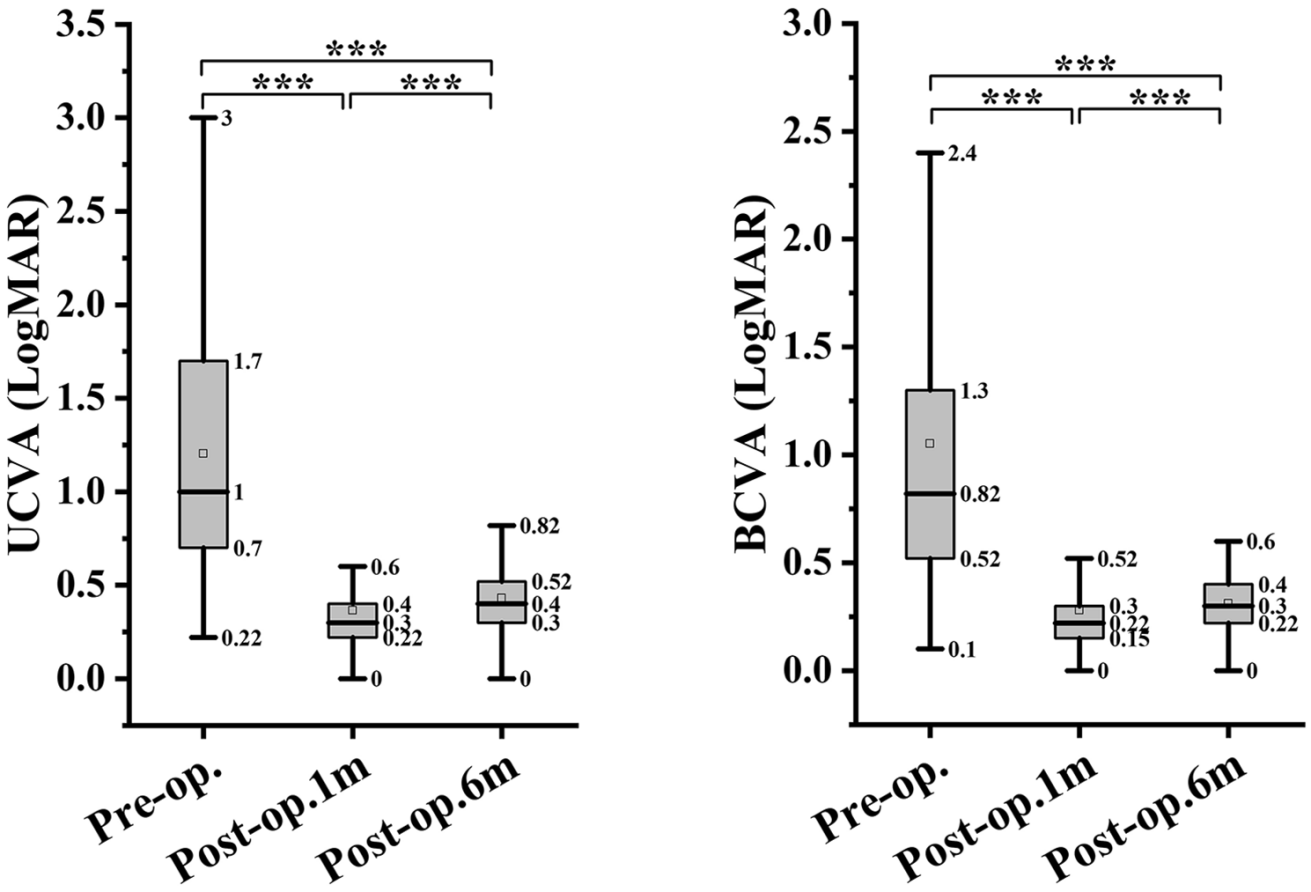
Comparison of UCVA and BCVA in the experimental group preoperatively, and at 1 month and 6 months postoperatively. UCVA, uncorrected visual acuity; BCVA, best corrected visual acuity; logMAR, logarithm of minimum angle of resolution; ***, *p* < 0.001

**Fig. 2:**
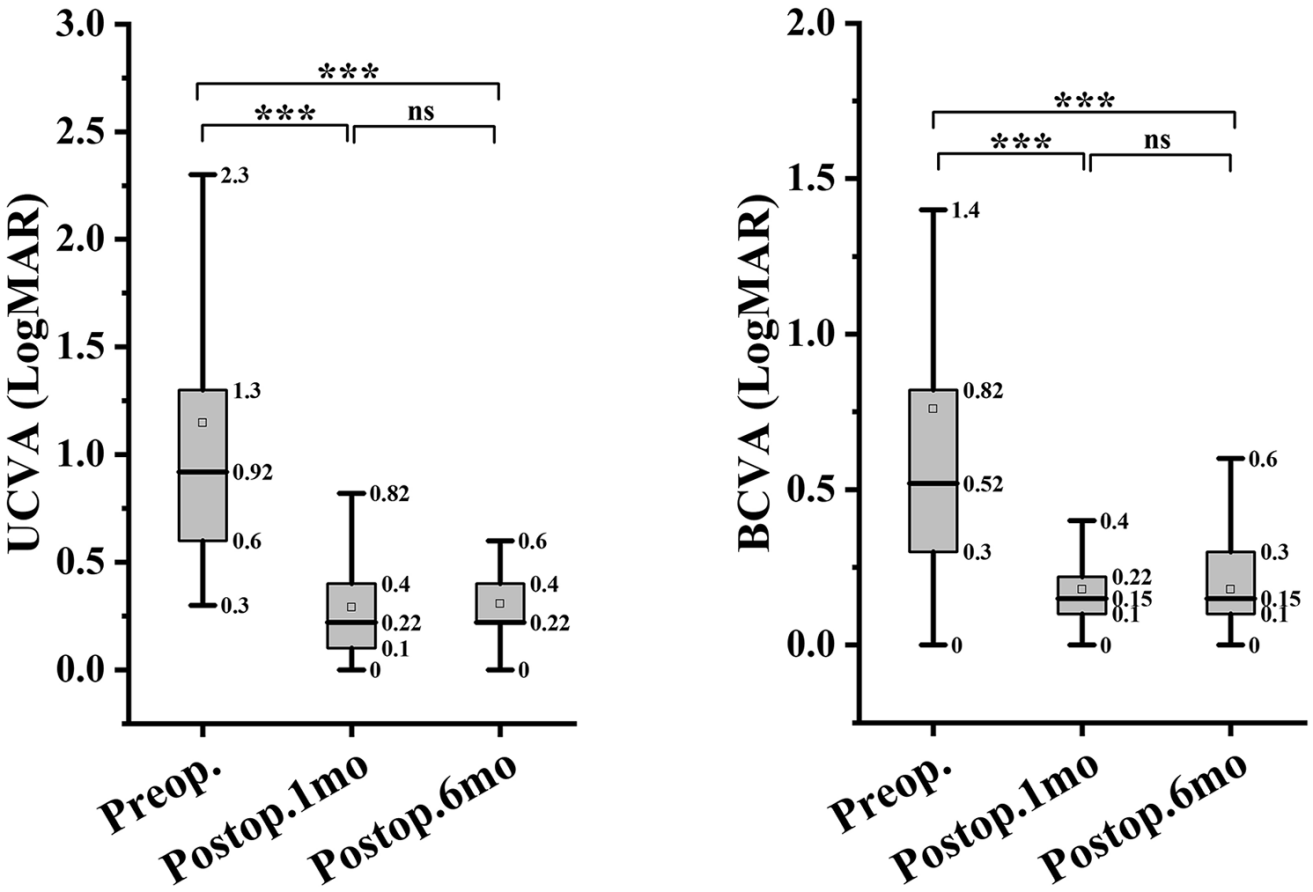
Comparison of UCVA and BCVA in the control group preoperatively, and at 1 month and 6 months postoperatively. UCVA, uncorrected visual acuity; BCVA, best corrected visual acuity; logMAR, logarithm of minimum angle of resolution; ***, *p* < 0.001; ns, not significant

**Fig. 3:**
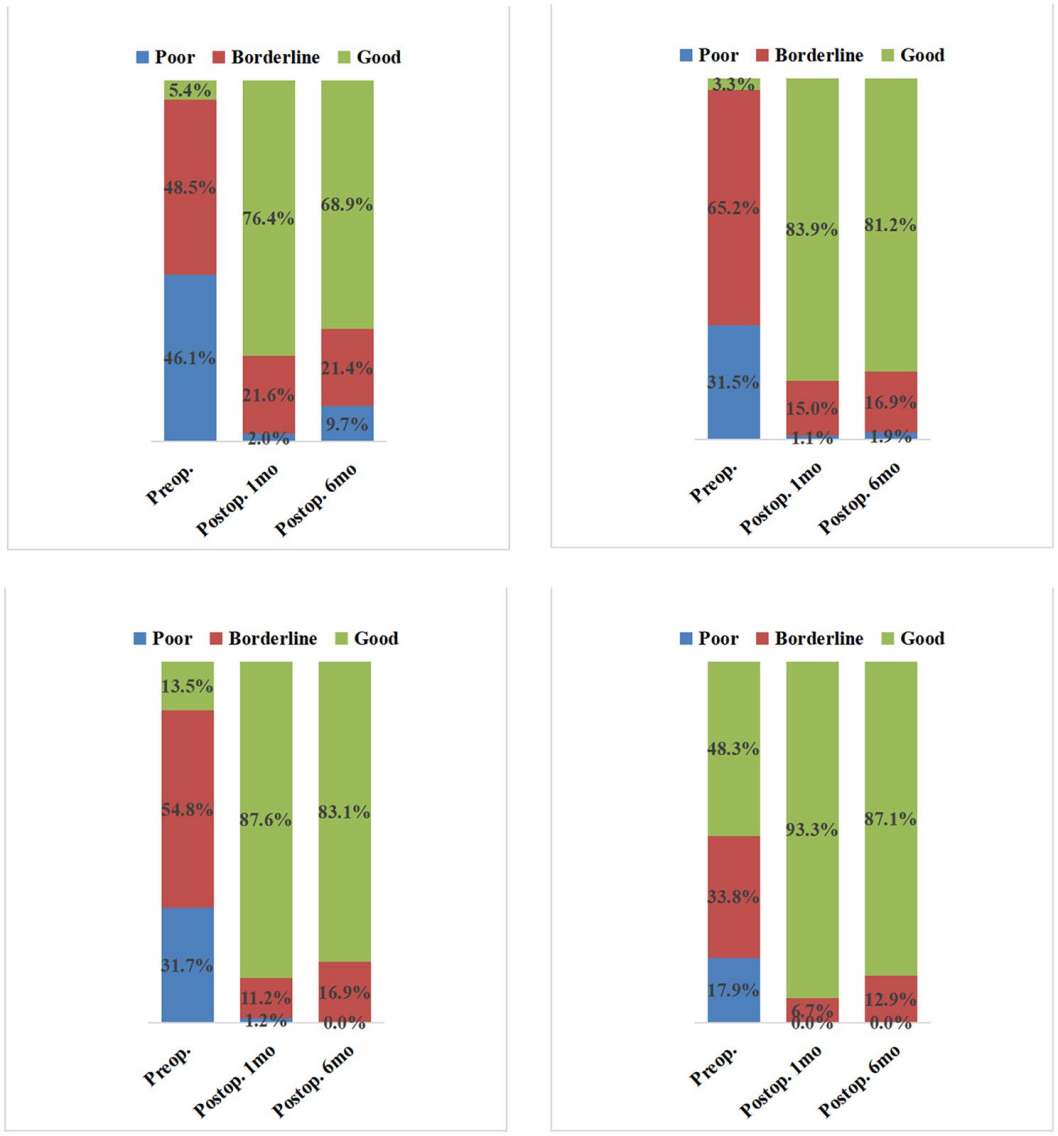
Distribution of UCVA and BCVA in the experimental and control groups preoperatively, and at 1 month and 6 months postoperatively. UCVA, uncorrected visual acuity; BCVA, best corrected visual acuity; good outcome, ≤ 0.5 logMAR; borderline outcome, > 0.5 logMAR to ≤ 1.0 logMAR; and poor outcome, > 1.0 logMAR

In both groups, the UCVA and BCVA at 1 month and 6 months postoperatively were significantly better than the corresponding preoperative values. The UCVA and BCVA were significantly worse at 6 months postoperatively than at 1 month postoperatively in the experimental group (*P* < 0.001; Figs. 1 and 2), while no significant difference in the postoperative values was found in the control group (UCVA, *P* = 0.057 and BCVA, *P* = 0.583; Fig. 2). The preoperative UCVA and BCVA significantly differed between the two groups, so the difference in the postoperative visual improvement between the two groups was not calculated. Figure 3 shows the distribution of UCVA and BCVA in the experimental and control groups before the operation, and at 1 month and 6 months after the operation. The rates of good outcomes of UCVA and BCVA in the experimental group were 76.2% and 87.6%, respectively, at 1 month postoperatively and 68.9% and 83.1%, respectively, at 6 months postoperatively.

### VF-QOL questionnaire scores

According to the multiple comparison of the VF-QOL questionnaire scores at each study time point between the two groups, we found that the postoperative scores (at 1 month and 6 months) were higher than the preoperative scores in both groups (*P* < 0.001; Table 3). However, the VF-QOL scores in the experimental group and the QOL score in the control group were significantly lower at 6 months than at 1 month postoperatively (*P* < 0.01; Table 3); the VF score at 6 months did not significantly differ from the VF score at 1 month in the control group (*P* = 0.20; Table 3).

**Table 3:** Comparison of VF-QOL questionnaire scores before the surgery and at 1 month and 6 months after the surgery

Score	Baseline	1 month	6 months	*P1*	*P2*	*P3*
**Experimental group**						
**VF**	48.48 (30.30, 60.61)	90.91 (84.85, 93.94)	87.88 (81.82, 93.94)	< 0.001	< 0.001	0.005
**QOL**	75.00 (61.11, 86.11)	100.00 (97.22, 100.00)	97.22 (94.44, 100.00)	< 0.001	< 0.001	< 0.001
**Control group**						
**VF**	48.48 (27.27, 57.58)	90.91 (87.88, 93.94)	90.91 (84.85, 93.94)	< 0.001	< 0.001	0.200
**QOL**	75.00 (63.89, 83.33)	100.00 (100.00, 100.00)	97.22 (97.22, 100.00)	< 0.001	< 0.001	< 0.001

Kruskal-Wallis *H* test

*P1*, preoperative vs. 1 month postoperatively

*P2*, preoperative vs. 6 months postoperatively

*P3*, 1 month vs. 6 months postoperatively

VF, visual function; QOL, quality of life

The VF and QOL scores were significantly lower in the experimental group than in the control group at 1 month and 6 months postoperatively (*P* < 0.05; Table 4).

**Table 4:** Comparison of VF-QOL questionnaire scores in the experimental and control groups at 1 month and 6 months postoperatively

Score	Experimental group	Control group	*Z* value	*P* value^*^
**1 month postoperatively**				
**VF**	90.91 (84.85, 93.94)	90.91 (87.88, 93.94)	3.332	0.001
**QOL**	100.00 (97.22, 100.00)	100.00 (100.00, 100.00)	2.761	0.006
**6 months postoperatively**				
**VF**	87.88 (81.82, 93.94)	90.91 (84.85, 93.94)	5.175	< 0.001
**QOL**	97.22 (94.44, 100.00)	97.22 (97.22, 100.00)	3.233	0.001

^*^ Mann-Whitney *U* test

VF, visual function; QOL, quality of life

### Refractive status

The refractive status was expressed as the spherical equivalent (SE). We found that at 1 month postoperatively, the refractive status was more dispersed in the experimental group than in the control group (*P* = 0.007; Table 5). The proportion of patients with refractive status < -1.0 D was higher in the experimental group than in the control group (13.5% vs. 7.8%, *P* < 0.05; Table 5), and the proportion of patients with refractive status between − 1.0 D and 1.0 D was lower in the experimental group than in the control group (82.1% vs. 89.5%, *P* < 0.05; Table 5).

**Table 5:** Distribution of refractive status in the experimental and control groups at 1 month postoperatively

Group	Total (eyes)	< -1.0 D [eyes (%)]	-1.0 to 1.0 D [eyes (%)]	> 1.0 D [eyes (%)]	*χ* ^*2*^	*P* ^*#*^
**Experimental**	453	61 (13.5%)	372 (82.1%)	20 (4.4%)	10.02	0.007
**Control**	447	35 (7.8%)^*^	400 (89.5%)^*^	12 (2.7%)		

D, diopter

^*^P < 0.05 vs. experimental group

^#^Chi squared test

### Incidence of PCO

In both groups, the incidence of PCO was significantly higher at 6 months than at 1 month (experimental group, 20.9% vs. 5.7%, *P* < 0.001; control group, 15.0% vs. 4.3%, *P* < 0.001). The incidence of PCO at 6 months was significantly higher in the experimental group than in the control group (20.9% vs. 15.0%, *P* = 0.022), but that at 1 month did not differ between the two groups (5.7% vs. 4.3%, *P* = 0.244).

### Causes of visual impairment

Table 6 shows the causes of visual impairment, which was defined as a UCVA worse than 0.5 logMAR at 6 months postoperatively. The main causes of visual impairment in the experimental group and the control group were uncorrected refractive errors (33.0% vs. 42.9%), PCO (29.5% vs. 25.7%), and fundus diseases (33.9% vs. 22.9%), and their incidence did not significantly differ between the two groups (*P* = 0.166).

**Table 6:** Causes of UCVA worse than 0.5 logMAR in the experimental and control groups at 6 months postoperatively

	Experimental group [eyes (%)]	Control group [eyes (%)]
Refractive error	37 (33.0%)	30 (42.9%)
Posterior capsule opacification	33 (29.5%)	18 (25.7%)
Fundus diseases	38 (33.9%)	16 (22.9%)
Age-related macular degeneration	9	3
Diabetic retinopathy	11	3
Retinal detachment	3	2
Macular epiretinal membrane	4	2
Macular hole	2	0
Retinal vein occlusion	3	1
Other retinopathy	4	3
Optic atrophy	2	2
Others	4 (3.6%)	6 (8.6%)
IOL tilt or decentration	3	3
Uncertain	1	3
Total	112	70

UCVA, uncorrected visual acuity; logMAR, logarithm of minimum angle of resolution; IOL, intraocular lens

## Discussion

We conducted a prospective comparative study to assess the long-term efficacy of cataract surgeries performed during blindness prevention programs in district and county hospitals vs. that performed during routine clinical practice in tertiary teaching hospitals in Chongqing, China. In our study, cataract surgery performed during blindness prevention programs significantly improved the patients’ VA, VF, and QOL, and the rates of good outcomes of UCVA and BCVA at 1 month after the surgery were 76.2% and 87.6%, respectively, which were almost equal to the WHO recommendation [23]. However, the incidence of PCO and refractive error were higher than those in the group that underwent cataract surgery in the tertiary teaching hospital. The main causes of postoperative visual impairment in both groups were uncorrected refractive errors, PCO, and fundus diseases. The overall efficacy of cataract surgeries performed during blindness prevention programs in Chongqing, China was satisfactory.

We did not compare the outcome of VA due to the significant difference in the preoperative VA between the two groups. In our study, the good outcome rates of UCVA and BCVA in the blindness prevention program group were 76.2% and 87.6% at 1 month postoperatively, which is comparable to the rates found in Beijing (79.7%) [24] and Chongqing, China (74.3%) [6], but higher than the rates reported in studies conducted in urban southern China (62.2%) [25], rural southwestern China (65.9%) [26], Nigeria (69%) [27], southwest Ethiopia (70.4%) [28], and India (64%) [29]. Although this result was partly attributable to differences in study populations and surgical procedures, it is sufficient to demonstrate a good outcome.

Studies have indicated that the mean scores on VF-QOL questionnaires are directly correlated with vision status [30, 31]. In our study, similar to the outcome of VA, the VF-QOL questionnaire scores were significantly improved postoperatively, and were better than the scores reported in studies from Hong Kong, Shunyi, Doumen, and Eastern China [31–34]. Although the preoperative VA was significantly worse in the blindness prevention program group than in the conventional cataract surgery group, there was no significant difference in the preoperative VF-QOL questionnaire scores. Akpolat et al. [35] reported that cataract patients with a higher education level had worse vision-related QOL than those with a lower education level, even if the baseline BCVA was similar. Munaw and Tegegn [36] reported that participants with high educational levels who are visually impaired are twice as likely to develop psychological distress than those who cannot read or write. In our study, most of the patients in the blindness prevention program group lived in rural areas and had a lower education level than that of the subjects in the conventional cataract surgery group. The differences in education level and lifestyle may explain the different preoperative VA but similar VF-QOL questionnaire scores in the two groups. At both 1 month and 6 months postoperatively, the blindness prevention program group exhibited significantly lower VF-QOL questionnaire scores than the conventional cataract surgery group. Furthermore, patients in the conventional cataract group demonstrated comparable VA and VF questionnaire scores at 1 month and 6 months, whereas patients in the blindness prevention program group experienced a significant reduction in VA and VF questionnaire scores at the 6-month mark relative to 1 month postoperatively. These findings indicate that the long-term postoperative outcomes of the blindness prevention program group were inferior to those of the conventional cataract surgery group. The higher incidence of PCO in the blindness prevention program group compared to the conventional cataract surgery group at 6 months postoperatively may be a leading contributor, as PCO is widely recognized as an important factor affecting long-term visual quality after cataract surgery.

PCO is the most common complication of cataract surgery, and it limits the long-term postoperative visual outcome. In our study, the incidence of PCO at 6 months postoperatively was 20.9% in the blindness prevention program group, which is lower than that reported by Gu et al. [37] (29.93% at 3 months postoperatively), but higher than that reported by Congdon et al. [22] (16.7% at 1 year postoperatively) and Ursell et al. [38, 39] (2.4–12.6% at 3 years and 5.8–19.3% at 5 years postoperatively). The incidence of PCO increases over time, so it is speculated that the incidence of PCO in our study may be higher than that in the studies conducted by Fong et al. [40] and Chassain and Chamard [41] (38.5% and 34% at 3 years postoperatively, respectively). The occurrence of PCO is related to many factors. Studies have suggested that the material and design of the IOL and the technique of capsular polishing are closely related to the development of PCO [42–44]. In our study, an additional hydropolish technique was performed in the conventional cataract surgery group, which is thought to be effective in reducing the incidence of PCO [21, 45]. In addition, although no detailed records were available, a hydrophobic acrylic lens was predominantly used in the conventional cataract surgery group, whereas a hydrophilic acrylic lens was predominantly in the blindness prevention program group. These differences may have contributed to the different rates of PCO between the 2 groups at 6 months after the surgery.

Residual refractive error is an important factor affecting the recovery of postoperative VA in patients with cataract [46]. The majority of the eyes in the blindness prevention program group (82.1%) and in the conventional cataract surgery group (89.5%) had a refractive error of within ± 1 D at 1 month postoperatively. However, the proportion of patients with a refractive status < -1.0 D at 1 month after the surgery was significantly higher in the blindness prevention program group (13.5%) than in the conventional cataract surgery group (7.8%). We were unable to compare the prediction error of the IOL power calculation between the two groups due to a lack of detailed records of the target diopter. However, given that the target diopter was between − 0.5 D and 0 D most patients, it is reasonable to believe that more patients had a myopic refractive surprise in the blindness prevention program group than in the conventional cataract surgery group. Studies have shown that the accurate calculation of the IOL power is the key to predicting the postoperative refractive status, and the measurement of ocular biological parameters is the main factor affecting the accuracy of IOL power calculation [46, 47] Optical biometry has been shown to be more accurate and repeatable than ultrasound A-scan biometry [48, 49]. The measurement of a shorter axial length, caused by excessive pressure on the cornea, is one of the most important sources of error in ultrasonic biometry [47]. This error results in a postoperative myopic refractive surprise. In our study, the differences in ocular biometrics may have contributed to the different refractive errors after cataract surgery. Another possible reason is that a few patients in district and county hospitals may have received IOLs with inappropriate power because of the limitation of IOL selection.

Similar to previous findings [19, 25, 50, 51], the main causes of visual impairment (UCVA worse than 0.5 logMAR) in both groups of patients were uncorrected refractive errors, PCO, and fundus diseases at 6 months postoperatively, but the number of eyes with visual impairment was greater in the blindness prevention program group (112 eyes) than in the conventional cataract surgery group (70 eyes). Although patients with vision-threatening ocular diseases detected by preoperative examination were excluded from our study, fundus diseases were still an important cause of postoperative visual impairment. It is possible that due to the severity of the cataract, the fundus could not be adequately examined before the surgery. The incidence of visual impairment due to fundus diseases was higher in the blindness prevention program group (38 eyes) than in the conventional cataract surgery group (16 eyes), which may be related to the lack of fundus examination equipment in district and county hospitals, allowing more fundus diseases to go undetected before the surgery.

In our study, patients who underwent cataract surgery during blindness prevention programs had poorer preoperative vision and a higher proportion of hard nuclear cataracts than patients in the conventional cataract surgery group. Compared with patients in the conventional cataract surgery group, the majority of the patients in the blindness prevention program group lived in rural areas, had a lower education level, and were more likely to be affected by issues related to transportation, economic conditions, and medical resources, resulting in more patients who did not choose to seek medical care until vision loss had severely affected their lives. Interestingly, studies have reported that women have a higher prevalence of cataracts and lower cataract surgery coverage than men [6, 52, 53], but in our study, the proportion of women in both groups was higher than that of men, which may indirectly indicate that the treatment of cataract blindness in Chongqing has achieved remarkable results.

Our study has some limitations. First, the missing rate was high at 6 months post-cataract surgery, which may have caused bias in the research results. Second, the difference in preoperative VA between the two groups affected the comparison of the surgical outcomes. Finally, the follow-up time was short, so the results may not effectively reflect the long-term efficacy of cataract surgery.

## Conclusions

In conclusion, the VA, VF, and QOL were significantly improved after cataract surgery during blindness prevention programs in Chongqing, China, but there was still a gap in the surgical outcomes, as compared with the surgeries performed in the tertiary teaching hospital. The main causes of visual impairment after cataract surgery performed during blindness prevention programs in Chongqing were residual refractive errors, PCO, and fundus diseases.

## Data Availability

The datasets generated and/or analyzed during the current study are not publicly available due to ongoing follow-up studies, but are available from the corresponding author on reasonable request.

## References

[1] GBD 2019 Blindness and Vision Impairment Collaborators; Vision Loss Expert Group of the Global Burden of Disease Study. Causes of blindness and vision impairment in 2020 and trends over 30 years, and prevalence of avoidable blindness in relation to VISION 2020: the right to Sight: an analysis for the global burden of Disease Study. Lancet Glob Health. 2021 Feb;9(2):e144–60. 10.1016/S2214-109X(20)30489-7 PMC7820391 33275949

[2] Xu TL, Wang BS,Liu H (2020). Prevalence and causes of vision loss in China from 1990 to 2019: findings from the global burden of Disease Study 2019.[J]. Lancet Public Health.

[3] Zhao JL, Xu X, Ellwein LB (2019). Causes of visual impairment and blindness in the 2006 and 2014 Nine-Province surveys in Rural China[. J] Am J Ophthalmol.

[4] Song P, Wang H,Theodoratou E (2018). The national and subnational prevalence of cataract and cataract blindness in China: a systematic review and meta-analysis[. J] J Glob Health.

[5] He MG, Wang W, Zhao. JL,[Prevention of blindness and ophthalmic epidemiology in China over the past 70 years].[J].Zhonghua Yan Ke Za Zhi, 2020, 56: 561–6. 10.3760/cma.j.cn112142-20200602-00366 32847332

[6] Zhao JL, Xu X, Ellwein LB (2018). Cataract Surgical Coverage and Visual Acuity Outcomes in Rural China in 2014 and comparisons with the 2006 China Nine-Province Survey. [J] Am J Ophthalmol.

[7] Mayinuer Y, Wang. NL,[Vision 2020: the progress of blindness prevention and eye health in China].[J].Zhonghua Yi Xue Za Zhi, 2020, 100: 3831–3834. 10.3760/cma.j.cn112137-20200825-02468 33371626

[8] Daien Vincent,Le Pape Annick,Heve Didier, et al. Incidence and Characteristics of Cataract Surgery in France from 2009 to 2012: A National Population Study.[J].Ophthalmology et al. 2015, 122: 1633-8. 10.1016/j.ophtha.2015.04.017 26050539

[9] Vs Murthy G, Jain B, Shamanna B (2014). Improving cataract services in the indian context. Community Eye Health.

[10] Elbieh Islam,Bascaran Covadonga,Blanchet Karl. Trends in cataract surgical rate and resource utilisation in Egypt.[J].Ophthalmic Epidemiol, 2018, 25: 351–7. 10.1080/09286586.2018.1481983 29883243

[11] Hashemi H et al. Fotouhi Akbar,Rezvan Farhad,. Cataract surgical rate in Iran: 2006 to 2010.[J].Optom Vis Sci, 2014, 91: 1355-9. 10.1097/OPX.0000000000000389 25237765

[12] Liu P, Zou HD, Hu AL (2017). [Critical elements of cataract prevention work in China].[J]. Zhonghua Yan Ke Za Zhi.

[13] Barraquer-Compte E, Rocha-de-Lossada C, Ferreiro-Vazquez T, Valvecchia G, Fernández J. Logistics description of a high yield cataract surgery non-profitable expedition. Arch Soc Esp Oftalmol (Engl Ed). 2023 Apr;98(4):193–8. 10.1016/j.oftale.2022.12.002 36801255

[14] Feng JJ, An L, Zhan LL (2019). Analysis on the ophthalmic medical service ability of public general hospitals at county level in China [J]. Chin J Strabismus Pediatr Ophthalmol.

[15] Feng JJ, An L, Wang ZF (2018). [Analysis on ophthalmic human resource allocation and service delivery at county level in Mainland China in 2014].[J]. Zhonghua Yan Ke Za Zhi.

[16] An L, Jan CL, Feng JJ et al. Inequity in Access: Cataract Surgery Throughput of Chinese Ophthalmologists from the China National Eye Care Capacity and Resource Survey.[J].Ophthalmic Epidemiol, 2020, 27: 29–38. 10.1080/09286586.2019.1678654 31635501

[17] Wu X, Shi X, Li H et al. Temporal and spatial characteristics of cataract surgery rates in China. Risk Manag Healthc Policy 2021 Aug 25;14:3551–61. 10.2147/RMHP.S317547 PMC8405972 34475788

[18] Fletcher AE, Ellwein LB, Selvaraj S (1997). Measurements of vision function and quality of life in patients with cataracts in southern India. Rep instrument Dev [J] Arch Ophthalmol.

[19] Javaloy, Jaime et al. Moya Tomás,Muñoz Gonzalo,. Efficacy, safety and visual outcomes of cataract surgeries performed during blindness prevention programs in different locations in Kenya.[J].Graefes Arch Clin Exp Ophthalmol, 2021, 259: 1215–1224. 10.1007/s00417-021-05084-5 33512611

[20] Limburg H, Foster A, Gilbert C (2005). Routine monitoring of visual outcome of cataract surgery. Part 1: development of an instrument[. J] Br J Ophthalmol.

[21] Wang Sarah B, Quah Xhian M, Amjadi Shahriar (2015). Hydropolish: a controlled trial on a technique to eradicate residual cortical lens fibers in phacoemulsification cataract surgery[. J] Eur J Ophthalmol.

[22] Congdon N, Fan H, Choi K (2008). Impact of posterior subcapsular opacification on vision and visual function among subjects undergoing cataract surgery in rural China: study of cataract outcomes and Up-Take of services (SCOUTS) in the Caring is Hip Project, report 5.[J]. Br J Ophthalmol.

[23] World Health Organization (1998). Informal Consultation on analysis of blindness Prevention Outcomes.

[24] Liu B, Xu L, Wang YX, et al. Prevalence of cataract surgery and postoperative visual outcome in Greater Beijing: the Beijing Eye Study. Ophthalmology. 2009 Jul;116(7):1322–31. 10.1016/j.ophtha.2009.01.030 19500855

[25] Huang W, Huang G, Wang D et al. Outcomes of cataract surgery in urban southern China: the Liwan Eye Study. Invest Ophthalmol Vis Sci 2011 Jan 5;52(1):16–20. 10.1167/iovs.10-5382 PMC3053272 20688728

[26] Shen W, Yang Y, Yu M (2013). Prevalence and outcomes of cataract surgery in adult rural chinese populations of the Bai nationality in Dali: the Yunnan minority eye study. PLoS ONE.

[27] Mpyet C, Langnap L, Akpan S. Outcome and benefits of small incision cataract surgery in Jos, Nigeria. Niger J Clin Pract. 2007 Jun;10(2):162–5. 17902511

[28] Addisu Z, Solomon B. Patients’ preoperative expectation and outcome of cataract surgery at jimma university specialized hospital -department of ophthalmology. Ethiop J Health Sci. 2011 Mar;21(1):47–55. 10.4314/ejhs.v21i1.69044 PMC3275855 22434985

[29] Nangia V, Jonas JB, Gupta R, et al. Prevalence of cataract surgery and postoperative visual outcome in rural central India Central India Eye and Medical Study. J Cataract Refract Surg. 2011 Nov;37(11):1932–8. 10.1016/j.jcrs.2011.08.020 21908172

[30] Fletcher AE, Ellwein LB, Selvaraj S, et al. Measurements of vision function and quality of life in patients with cataracts in southern India. Report of instrument development. Arch Ophthalmol. 1997 Jun;115(6):767–74. 10.1001/archopht.1997.01100150769013 9194729

[31] Zhou JB, Guan HJ, Zhu DQ (2013). Improvement in visual function and quality of life following a blindness prevention surgery program in a rural area of Eastern China. [J] Exp Ther Med.

[32] Zhao J, Sui R, Jia L (1998). Visual acuity and quality of life outcomes in patients with cataract in Shunyi County. China [J] Am J Ophthalmol.

[33] He M, Xu J, Li S (1999). Visual acuity and quality of life in patients with cataract in Doumen County. China [J] Ophthalmology.

[34] Lau J, Michon JJ, Chan WS, et al. Visual acuity and quality of life outcomes in cataract surgery patients in Hong Kong. Br J Ophthalmol. 2002 Jan;86(1):12–7. 10.1136/bjo.86.1.12 PMC1770956 11801495

[35] Akpolat C, Demir M, Cevher S et al. The impact of phacoemulsification surgery on vision-related quality of life in senile cataract patients. Ther Adv Ophthalmol. 2022 Jan 21;14:25158414211063293. 10.1177/25158414211063293 PMC8785351 35083419

[36] Munaw MB, Tegegn MT. Visual impairment and psychological distress among adults attending the University of Gondar tertiary eye care and training center, Northwest Ethiopia: a comparative cross-sectional study. PLoS One 2022 Feb 17;17(2):e0264113. 10.1371/journal.pone.0264113 PMC8853488 35176097

[37] Gu X, Chen X, Jin G, et al. Early-onset posterior Capsule Opacification: incidence, severity, and risk factors. Ophthalmol Ther. 2022 Feb;11(1):113–23. 10.1007/s40123-021-00408-4 PMC8770765 34727350

[38] Ursell PG, Dhariwal M, Majirska K et al. Three-year incidence of nd:YAG capsulotomy and posterior capsule opacification and its relationship to monofocal acrylic IOL biomaterial: a UK Real World evidence study.[J].Eye (Lond), 2018, 32: 1579–89. 10.1038/s41433-018-0131-2 PMC6189124 29891902

[39] Ursell PG, Dhariwal M et al. 5 year incidence of YAG capsulotomy and PCO after cataract surgery with single-piece monofocal intraocular lenses: a real-world evidence study of 20,763 eyes.[J].Eye (Lond), 2020, 34: 960–8. 10.1038/s41433-019-0630-9 PMC7182577 31616057

[40] Fong CS, Mitchell P, Rochtchina E (2014). Three-year incidence and factors associated with posterior capsule opacification after cataract surgery: the australian prospective cataract surgery and age-related Macular Degeneration Study[. J] Am J Ophthalmol.

[41] Chassain C (2018). Chamard C,[Posterior capsule opacification, glistenings and visual outcomes: 3 years after implantation of a new hydrophobic IOL]. [J] J Fr Ophtalmol.

[42] Zhang Y, Zhang C, Chen S et al. Research Progress concerning a novel intraocular Lens for the Prevention of posterior capsular opacification. Pharm 2022 Jun 25;14(7):1343. 10.3390/pharmaceutics14071343 PMC9318653 35890240

[43] Kwon YR, Hwang YN, Kim SM. Posterior Capsule Opacification after cataract surgery via implantation with Hydrophobic Acrylic Lens compared with silicone intraocular Lens: a systematic review and Meta-analysis. J Ophthalmol. 2022 Feb 25;2022:3570399. 10.1155/2022/3570399 PMC8896947 35251708

[44] Darian-Smith E, Safran SG, Coroneo MT. Lens epithelial cell removal in routine phacoemulsification: is it worth the Bother? Am J Ophthalmol. 2022 Jul;239:1–10. 10.1016/j.ajo.2022.01.013 35081415

[45] Liu Z, Cao Q, Qu B, et al. Fluid-jet technique to polish the posterior capsule for phacoemulsification surgeries: efficacy and safety evaluation. J Cataract Refract Surg. 2020 Nov;46(11):1508–14. 10.1097/j.jcrs.0000000000000319 32675653

[46] Khoramnia R, Auffarth G, Łabuz G et al. Refractive outcomes after cataract surgery. Diagnostics (Basel). 2022 Jan 19;12(2):243. 10.3390/diagnostics12020243 PMC8870878 35204334

[47] Norrby S (2008). Sources of error in intraocular lens power calculation[. J] J Cataract Refract Surg.

[48] Rajan MS, Keilhorn I, Bell JA. Partial coherence laser interferometry vs conventional ultrasound biometry in intraocular lens power calculations. Eye (Lond). 2002 Sep;16(5):552–6. 10.1038/sj.eye.6700157 12194067

[49] Landers J, Goggin M. Comparison of refractive outcomes using immersion ultrasound biometry and IOLMaster biometry. Clin Exp Ophthalmol. 2009 Aug;37(6):566–9. 10.1111/j.1442-9071.2009.02091.x 19702705

[50] Shen W, Cun Q, Zhong H (2020). Ethnic variation in prevalence, self-reported barriers and outcome of cataract surgery in a rural population in southwestern China: the Yunnan minority eye study[. J] BMC Public Health.

[51] Marmamula S, Khanna RC, Shekhar K et al. Outcomes of cataract surgery in Urban and Rural Population in the South Indian State of Andhra Pradesh: Rapid Assessment of Visual Impairment (RAVI) Project. PLoS One 2016 Dec 5;11(12):e0167708. 10.1371/journal.pone.0167708 PMC5137898 27918589

[52] Ramke J, Zwi AB, Lee AC, et al. Inequality in cataract blindness and services: moving beyond unidimensional analyses of social position. Br J Ophthalmol. 2017 Apr;101(4):395–400. 10.1136/bjophthalmol-2016-309691 28228412

[53] Lewallen S, Mousa A, Bassett K, et al. Cataract surgical coverage remains lower in women. Br J Ophthalmol. 2009 Mar;93(3):295–8. 10.1136/bjo.2008.140301 19091848

